# Evolving perspectives of medial temporal memory function: hippocampal processes in visual and auditory forms of episodic and working memory

**DOI:** 10.3389/fcogn.2024.1497281

**Published:** 2024-12-13

**Authors:** Chris Hawkins, Andrew P. Yonelinas

**Affiliations:** Department of Psychology, University of California, Davis, Davis, CA, United States

**Keywords:** working memory, familiarity, recollection, hippocampus, visual working memory, auditory working memory, medial temporal lobe, amnesia

## Abstract

A cornerstone of memory science is the finding that the medial temporal lobe plays a critical role in supporting episodic long-term memory. However, the role that this brain region plays in supporting other forms of memory such as working memory is controversial. In this selective review, we describe some of the key studies that have informed our current understanding of the role that the medial temporal lobe plays in working memory. We first describe the early studies that supported the idea that the medial temporal lobe is selectively important for long-term episodic memory function, then discuss the subsequent research that indicated that the hippocampus also plays a critical role in visual perception and visual working memory. We then review more recent work suggesting that the medial temporal lobe, and particularly the hippocampus, is critical in supporting a familiarity-based memory signal in working memory, and we propose that this function may not be limited to the visual domain, but rather may support familiarity for auditory working memory as well.

## 1 Introduction

Canonical studies of memory in humans and non-human primates suggest that the medial temporal lobe (MTL) and particularly the hippocampus (HC) plays a central role in supporting long-term memory (LTM) function, while playing a negligible role in working memory (WM) and perception. The latter two processes were long presumed to be supported by various regions in the cortex, but recent evidence suggests that the hippocampus supports both WM and perception, in addition to LTM, albeit in different ways. Specifically, whereas the hippocampus appears to support a “recollection” signal in LTM, assisting in the recall of high-confidence, qualitative information, in WM the hippocampus supports a lower-confidence, “familiarity”-based signal. Importantly, much of the research on hippocampal function has focused on the visual domain, leaving open questions about whether the region also supports auditory WM in an analogous manner to visual WM. In the current paper, we propose that the predominant role of the hippocampus is to support a familiarity-matching signal in working memory, and that its role generalizes across sensory modalities (i.e., visual and auditory).

## 2 The medial temporal lobe and episodic memory function

Historically, findings from neuropsychological studies firmly established the hippocampus as being critical for LTM functioning, while playing little if any role in other cognitive functions, such as WM and perception (e.g., Baddeley and Warrington, 1970; Scoville and Milner, 1957; Zola-Morgan et al., 1994). For example, in a 1957 paper, Scoville and Milner described 10 patients who underwent temporal lobe resections. Included in this group was the case of H.M., a patient who underwent a bilateral temporal lobe resection to treat medically intractable epilepsy. After surgery, a battery of cognitive tests revealed no deficits to his abstract thinking, perception, or reasoning, but a severe long-term memory deficit was apparent in the sense that H.M.'s free recall was severely impaired, and he did not show any practice effects in associative learning tasks. In fact, Scoville and Milner reported that in 8 cases of bilateral hippocampal resection each patient showed a severe memory deficit of similar type. Their work made clear the importance of the MTL for long-term memory function. Subsequent observations of MTL amnesics made a compelling case that LTM and WM were supported by different structures. For instance, when given a sequence of 3-letter words, patients with MTL damage showed no impairments in recall at delays of 0–12 s compared with age and IQ-matched controls, further, when given a list of nouns and asked to freely recall as many words as possible patients were comparable to controls for immediate recall, but significantly impaired relative to controls at delays of 30 s (Baddeley and Warrington, 1970). In short, WM and LTM were found to be decidedly distinct (Atkinson, 1968).

More recent work has indicated that different structures within the medial temporal lobe play distinct roles in supporting different forms of LTM. Most notably, patients with selective hippocampal damage are impaired at tasks requiring recollection of prior events, whereas damage to the surrounding perirhinal cortex (PRC) leads to impairments in familiarity-based discriminations. Recollection refers to high-confidence recognition judgments that are associated with the retrieval of qualitative information about the study event, such as where or when it occurred. Familiarity, by contrast, refers to judgments that can vary in confidence whereby an item is recognized as having been recently studied, but in the absence of an ability to recall where or when it was studied (for reviews see Yonelinas, 2023; Yonelinas et al., 2024). To examine the role of different MTL regions in supporting recollection and familiarity, Yonelinas et al. (2002) examined recall and recognition memory for studied words in mild hypoxic patients expected to have selective hippocampal damage and healthy controls, and they found that the patients exhibited significantly greater deficits in recall than in recognition tests. Structural modeling of those results revealed that hypoxia was related to a selective deficit in recollection that left familiarity-based discriminates entirely unaffected. In addition, they compared recognition performance the mild hypoxic group and in patients with damage to the hippocampus and surrounding parahippocampal gyrus (i.e., the MTL group), as well as age-matched controls. Compared to controls, both patient groups showed significantly reduced reports of remembering study details, however, the MTL group showed an additional deficit in reports of familiarity-based recognition. A final experiment examined recognition confidence rating receiver operating characteristics (ROC), which were used to derive parameter estimates of recollection and familiarity. Examination of the ROCs confirmed and extended their prior findings in showing that patients with damage restricted to the hippocampus were impaired in recollection, but not familiarity, whereas patients with damage to the hippocampus and surrounding MTL, exhibited deficits in both recollection and familiarity. Critically, the two patient groups showed comparable overall recognition memory performance, it was only when broken down into estimates of R and F that these behavioral dissociations presented. Thus, their results showed that long-term memory is supported by two processes, recollection and familiarity, which appear to be functionally localized to the hippocampus and PRC, respectively (Yonelinas et al., 2002).

A number of subsequent lesion studies in humans and rats have verified that the hippocampus is critical for recollection (e.g., Aggleton et al., 2005; Bastin et al., 2004; Bowles et al., 2010; Brandt et al., 2009; Düzel et al., 2001; Gilboa et al., 2006; Holdstock et al., 2002; Jäger et al., 2009; Mayes et al., 2002; Fortin et al., 2004; Montaldi et al., 2006; Turriziani et al., 2008; but see Squire et al., 2007). However, there are some patients who appear to have selective hippocampal lesions who exhibit deficits in both recollection and familiarity. For example, one mixed etiology group of patients consistently shows deficits in recollection and familiarity as well as equally severe deficits in recognition and recall tests (e.g., Kirwan et al., 2010; Reed and Squire, 1997; Manns and Squire, 1999; Manns et al., 2003). The impairments in that group could be a consequence of the specific subject selection protocol that was used to identify those patients, as they were selected to have extremely low scores on standardized memory measures that included both recall and recognition tests, and the patients exhibited unusually severe delayed memory scores (i.e., more than five standard deviations below normal in six of the seven patients; Yonelinas et al., 2004). The impairments of course could also be due to additional undetected brain damage. In fact, a recent study (Argyropoulos et al., 2022) reported recollection and familiarity deficits in a group of encephalitis patients with reduced hippocampal volume along with no significant reduction in PHC volume. Importantly, however, the recollection deficits were found to be related to hippocampal volume reductions and were not related to PHC volume, whereas familiarity was related to reductions in PHC volume but was unrelated to hippocampal volume. In sum, selective hippocampal damage generally leads to selective recollection impairments, but subtle damage to surrounding MTL cortex that may not be significant in individual patients can lead to familiarity impairments. The results are consistent with studies in rats in which controlled lesions to the hippocampus are found to lead to selective recollection impairments using ROC test procedures that parallel those used in human studies (Fortin et al., 2004). In addition, other human and rodent studies have indicated that damage to MTL regions outside the hippocampus such as the PRC can lead to deficits in familiarity that do not impair recollection (e.g., Bowles et al., 2007; Brown and Aggleton, 2001; Robinson et al., 2010; for review, see Eichenbaum et al., 2007).

There is some evidence that the hippocampus may play a material-specific role in episodic memory, in the sense that it may be less important in supporting memory for novel faces than other materials. For example, Cipolotti et al. (2006) observed a single patient with selective hippocampal damage who exhibited impaired recognition for words, buildings, and landscapes, but spared recognition for unknown faces. In addition, a secondary analysis of published data from hippocampal patients who had been administered a standardized test of recognition for words and faces revealed that the patient group was significantly impaired on word recognition whereas their reduction in face recognition was not significant (Bird, 2017). Another study that directly contrasted recognition memory for faces and words found that hippocampal damage led to significantly greater deficits in word recognition than in face recognition (Aly et al., 2010). However, that study found that the patients were significantly impaired in face recognition, and that this was associated with a deficit in recollection rather than familiarity. The results were interpreted as indicating that face recognition relied more heavily on familiarity than does word recognition, suggesting that hippocampal damage was less disruptive of face recognition than recognition of other materials.

In contrast to the work examining the role of MTL regions to different forms of LTM, other work has indicated that regions outside the MTL such as the parietal and frontal lobes are critical for WM. For example, a classic study by Warrington and Shallice (1969) showed that verbal working memory relied on the lateral parietal cortex but was not critical for LTM. Specifically, Warrington and Shallice published observations of patient K.F., a man with severely impaired WM (a digit span of 1–2 items), but intact LTM, resulting from a left parietal subdural hematoma secondary to parietooccipital fracture. Numerous other patients with parietal lobe lesions have been identified that show similar deficits (Buchsbaum et al., 2011). In addition, lesions to the prefrontal cortex have also been found to lead to comparable deficits in WM (for a review see Funahashi and Kubota, 1994).

In addition to studies of verbal WM, studies examining visual WM also suggested that it was supported by regions outside the MTL including the frontal and parietal regions, seemingly exclusive of the medial temporal lobe. For example, in one seminal study, Goldman and Rosvold (1970) examined the effects of lesions to several frontal areas on performance of two spatial tasks. Rhesus monkeys were trained on a spatial delayed alternation task in which they were required to displace objects covering one of two food compartments in alternating fashion, and a conditional position response task in which animals were cued to one of two compartments containing food. Though both tasks test visuospatial ability, critically, the former task involved a delay and is considered to reflect a process like working memory in humans, while the latter did not (Goldman-Rakic et al., 1984; Goldman-Rakic, 1991). Monkeys were surgically lesioned in one of four regions: the principal sulcus of the PFC, the arcuate sulcus, the dorsolateral surface (not including the principal sulcus), or the pre-motor area. The results of their experiments showed that monkeys with lesions to the principal sulcus were unable to learn the delayed alternation task, but were unimpaired for the conditional response task, whereas monkeys with lesions to the arcuate sulcus showed impaired performance in the conditional response task, but exhibited only an impairment in their time to re-learn the delayed alternation task post-operatively, but were eventually able to re-learn the task to criterion. Thus, Goldman and Rosvold's work seemed to point to the dlPFC, specifically the principal sulcus, as the base of operations for visuospatial memory in monkeys.

In humans, visual working memory function has also localized to prefrontal regions rather than the MTL. For example, neuropsychological evidence came from two groups of amnesic patients: six with hippocampal damage and six with diencephalic damage due to Korsakoff's syndrome (Cave and Squire, 1992). In that study, four tests of non-verbal WM (i.e., retention, apprehension, manipulation of non-verbal stimuli, and WM for spatial location) were administered to the patient groups, along with one verbal WM task (i.e., repeated digit span). The patients with hippocampal damage performed as well as controls on the digit span task, and when their memory for spatial location was tested, they performed comparably to controls until the delay period reached 24 s, indicating that neither visual nor phonological WM were dependent on the hippocampal formation. In contrast, the Korsakoff's patients exhibited deficits on the digit span, and non-verbal WM (e.g., memory for dot locations) tasks, even at 0 s delays. Moreover, impairments in the Korsakoff's group were particularly pronounced when delay-period interference was introduced, suggesting that their deficits could be reasonably tied to the effects of associated frontal lobe dysfunction (e.g., increased distractibility), rather than a specific memory deficit. Additional testing of two amnesics with circumscribed diencephalic damage exclusive of the frontal lobe revealed normal performance on all tasks, lending support to the notion that increased distractibility, and not impaired memory, was responsible for impaired performance in the Korsakoff's patient group.

Subsequent neuroimaging work seemed to converge with those findings. For example, Jonides et al. (1993) showed participants an array of dots for 200 ms during PET scanning, and after a 3 s delay, probed their memory for the location of one of the dots on the screen before. Their results showed significantly increased right-lateralized activation in the prefrontal cortex, posterior parietal cortex, occipital cortex, and premotor cortex. The authors suggested a model of visual WM wherein a subject generates a mental image of the visual object at presentation using mechanisms in the occipital cortex, then mechanisms in the parietal cortex compute spatial location. Finally, during the retention interval the maintenance process is carried out by the prefrontal cortex. Converging fMRI results were obtained by Courtney et al. (1997), who examined perceptual and maintenance processing of face stimuli. In their task, participants viewed a face for 3 s, then held the image of that face in mind throughout an 8 s retention interval before a second face was presented, participants then responded whether the test face matched the original face. Their results demonstrated increased neural activity in the posterior lingual and fusiform gyri in response to general (i.e., non-selective) visual stimulation, consistent with the involvement of those regions in early visual processing. Three additional regions showed increased activation during the memory delay period: the posterior middle and inferior frontal gyri (Brodmann area 9/44), the inferior frontal gyrus toward the anterior portion of the insula (Brodmann area 45/47), and the anterior midfrontal gyrus (Brodmann area 46). Importantly, the three regions showed differential engagement during the delay period, with the posterior middle and inferior frontal gyri showing the least amount of increased delay-period activation, and the anterior midfrontal gyrus showing the greatest increase in delay-period activity. In sum, their results helped to distinguish between occipitotemporal perceptual processing regions, and a network of prefrontal areas responsible for the actively maintaining stimulus representations over brief delays. Finally, work by Cohen et al. (1997) scanning participants while they performed an n-back sequential letter task with memory loads varying between 0- and 3-back. The results of their experiment confirmed the earlier findings from Courtney et al. (1997), showing that the dorsolateral prefrontal cortex plays a central role in active maintenance of relevant information, such that, activation of the region increased as a function of increasing memory load, and was sustained throughout the trial.

Following the discoveries of Dr. Goldman-Rakic, localizing WM to the PFC in rhesus monkeys, research on the neural substrates of visual WM has suggested that hippocampal damage did not result in impairments to visual working memory, whereas damage to the frontal lobes produced reliable visual WM impairments. Neuroimaging studies in healthy humans expanded our understanding of visual WM function by showing that parietal and frontal regions support perceptual processing and active maintenance of items in visual WM, respectively. Furthermore, while WM function was localized to the PFC and parietal lobe, the MTL was established as a critical structure for LTM function, and within it, the hippocampus was shown to support recollection, whereas the PRC supports familiarity.

## 3 The medial temporal lobe and visual perception

Evidence for the involvement of MTL structures in perception was first reported in two experiments with macaque monkeys (Buckley and Gaffan, 1998). In those experiments, monkeys with lesions to the PRC made significantly more errors than controls both when identifying familiar objects that were presented from different viewpoints, and when they were embedded in complex scenes. In addition, Buckley et al. (2001) administered a series of oddity discrimination tasks for objects, colors, shapes, and faces in which monkeys were trained to select the unique stimulus from among similar stimuli (e.g., given a set of object images taken from different viewpoints, select the one image that depicted an object that was different from the others). Importantly, the stimuli in each trial were randomly assigned to target or lure with equal probability, thus, the solutions (i.e., picking out the oddball from similar stimulus pairs) were perceptually available but the monkeys had no way of predicting from memory which stimulus choice would lead to a reward. Monkeys with PRC damage were impaired at discriminating oddball objects when the discriminations were based on object-level discriminations (e.g., discriminating between different views of similar objects) and not when the discriminations were feature-based (e.g., discriminating between different colors or object sizes; Buckley et al., 2001).

Subsequent work in humans examined the role of PRC and the hippocampus in visual perception. Lee et al. (2005a) adapted their 2001 paradigm (described above) and across several experiments found that perceptual discrimination of objects and faces was impaired in patients with MTL lesions that included both the PRC and hippocampus, but observed no such impairment in patients with selective hippocampal lesions. In contrast, perceptual discrimination of scenes was impaired in both the MTL (hippocampus + PRC) and hippocampal lesion groups. Analogous findings from patients with either selective hippocampal, or broader MTL damage were reported by Lee et al. (2005b). Their study found that patients with selective hippocampal damage were impaired at discriminating the correct spatial scene (the one which most matched a target scene) from two scenes which contained varying amounts of feature overlap, while patients with damage to the hippocampus and PRC were significantly impaired at discriminating the correct stimulus (the one which shares the most features with the target stimulus) between pairs of scenes, objects, and faces with varying amounts of feature overlap; though both patient groups showed relatively preserved ability to make feature-based discriminations (e.g., based on color). These results were further supported by McCormick et al. (2017) who found that patients with hippocampal damage performed comparably to controls when detecting semantic violations of complex scenes (i.e., judging whether a scene was semantically possible or impossible), but were impaired when judging whether a scene was constructively possible or impossible (see McCormick and Maguire, 2021 for neuroimaging support of these results).

Thus, these results confirm and extend what was observed by Buckley et al. (2001), suggesting that the perception of complex objects and faces depended on the PRC, whereas the perception of complex scenes relied on the hippocampus. However, as we describe in the following section, working memory impairments in patients with hippocampal damage are not limited to spatial materials but rather generalize to a variety of other stimuli including simple gabor patches and novel objects (Goodrich et al., 2019; Yonelinas et al., 2024), suggesting that although the hippocampus plays an important role in processing scenes, it may be involved in the perception of other materials as well.

The above-described findings helped shape what we now know about medial temporal lobe function. First, contrary to what would have been anticipated, lesions to the primate PRC result in severe perceptual impairments as evidenced by impaired object discrimination performance which cannot be attributed to learning impairments. In addition, from studies of humans with hippocampus and broader MTL lesions, the results suggest that the human hippocampus and PRC may be particularly important for the perception of scenes and objects, respectively.

## 4 The medial temporal lobe and visual working memory

Consistent with the studies of visual perception that we just described, a number of studies suggested that the MTL may also play a critical role in visual working memory. For instance, Aggleton et al. (1992) examined rodents with hippocampal or fornix lesions using a delayed non-match to position task. In their study, rodents were first presented with a retractable lever in one of two possible spatial positions. After pulling the lever, it was retracted, and a delay period began. Following the delay period both levers were presented and the animal was rewarded if they selected the lever that had *not* been presented during the study phase. Their results showed that rodents with lesions to the hippocampus, as well as those with lesions to the fornix, were both impaired relative to control animals in the task, even at delays of < 4 s, suggesting that the hippocampus is necessary for the maintenance of visuospatial information over brief delays (Aggleton et al., 1992).

Soon after the observations of Aggleton et al., evidence for hippocampal involvement in human visual WM was also reported. In one early experiment, researchers used event-related fMRI to examine the role of the MTL in active maintenance during visual WM (Ranganath and D'Esposito, 2001). In that study, participants viewed trial-unique faces during fMRI scanning, and were asked to maintain a mental image of the face over a 7 s delay, after which, a probe face was presented, and participants judged whether it was the same as, or different from the original sample face. Participants also underwent an incidental encoding and recognition (LTM) task for novel faces under the same temporal parameters; thus, the latter task allows for the assessment of LTM-related activation in such a way as to rule out the possibility that any findings from the former task are related to LTM encoding. Analysis of the fMRI activations revealed increased delay-period activity in the anterior portion of the hippocampus, which was not observed for the encoding or retrieval phases in either the WM or LTM tasks. By contrast, voxels in the parahippocampal gyrus exhibited increased activation during the encoding and response phases of both the WM and LTM tasks. These results suggest that although the MTL is involved in encoding and retrieval of WM and LTM, the anterior hippocampus may be preferentially involved during WM maintenance.

A number of patient studies have also suggested that the MTL plays a critical role in visual WM. For example, across three experiments Olson et al. (2006a) tested whether visual working memory for locations, faces, or colors, was impaired at either 4 or 8 s delays in patients with MTL damage and age-matched controls. In experiment 1, participants had to remember 3 or 6 locations (i.e., squares), across a 4 s delay, after which a second screen appeared with one of the squares removed, and the participants had to determine which previously filled location was now missing. In a perceptual control task, there was no delay between the studied and tested images. Results of experiment 1 showed that MTL group had significantly impaired visual WM for locations relative to controls, even for the set size of 3, while they were not significantly different from controls on the perceptual control task. Experiment 2 and 3 examined memory for faces and colors, respectively, and indicated that MTL damage disrupted WM but did not disrupt performance on the perceptual control tasks.

Other studies, however, have indicated that not all forms of WM uniformly rely on the MTL. For example, a second study from the Olson group indicated that the MTL may be particularly important for maintaining memory for associations rather than simple items, and that the associative maintenance impairments were due to hippocampal damage, rather than damage to other MTL regions (Olson et al., 2006b). In two experiments, MTL amnesics and controls viewed 3 objects, presented sequentially at 3 of 9 possible locations on a grid, and after a 1 or 8 s delay made a recognition judgment (same or different) about either a probe object, location, or object-location conjunction. For example, in “object” trials, after the delay period, an image of one of the sequentially presented items (“same”) or a completely novel image (“different”) would appear in the center of the grid. For “object-location” trials, after the delay period, an image of one of the sequentially presented items would either appear in its originally presented location (“same”) or presented with an incorrect location (i.e., a location where a different item had originally been presented, not a novel location; “different”). Finally, in “location” trials, after the delay, a black circle appeared in the location of a previously presented item (“same”) or a new location not previously occupied (“different”). The results indicated that patients exhibited intact feature memory (i.e., memory for either objects or locations) relative to controls, but were severely impaired on memory for conjunctions (object + location). Comparable impairments were observed for patients with restricted hippocampal damage as well as those with larger MTL lesions, suggesting that the hippocampus is particularly important for associative WM. The results are broadly consistent with other studies showing hippocampal involvement in WM tasks that require memory for complex associations, such as object-location bindings and spatial configurations (Hannula et al., 2006; Hartley et al., 2007).

Taken together, the pattern of results described above suggest that the hippocampus may not be particularly critical for the maintenance of simple item information. Rather, the hippocampus and MTL may be concerned primarily with the binding of stimulus *associations*, even across very brief delays.

It is important to note that the role of the hippocampus in binding is somewhat controversial. For example, Baddeley et al. (2010) used a change detection task to examine both visual object and sentence binding in WM in a patient, Jon, who suffered severe perinatal hippocampal damage. Three binding conditions were assessed: colored shapes, “spatially separated” shapes (i.e., colored splotch + uncolored shape), and cross-modal shapes (i.e., shape + aurally presented color words). Participants viewed a sequence of paired stimuli (e.g., color + shape) at varying set sizes, and after a brief delay were presented with a probe stimulus. Participants made a binary response indicating whether the probe was present in the preceding study array or not. If the hippocampus is critical for binding stimulus associations, then, relative to controls, Jon's performance should be impaired for all three binding conditions. Contrary to that prediction, Jon showed spared performance on each task. In fact, Jon outperformed younger control subjects on each binding task, in one condition (“spatially separated”) by as much as one standard deviation above the control group average. In the word/sentence binding task, participants saw sequences of words (i.e., meaningful sentences) and word lists of varying length with and without the use of articulatory suppression (that is, repeating an irrelevant word to prevent subvocal rehearsal). Jon's performance in the sentence binding was again comparable to controls, suggesting that hippocampal damage is not sufficient to produce binding deficits.

These results are difficult to reconcile in the context of the findings discussed earlier. Why did Baddeley and colleagues fail to find evidence for binding across so many task types? One explanation could be that the results may be specific to that one patient who was unique in the sense that his IQ is higher than average, and his overall cognitive functioning is well-preserved and in some cases superior to controls. Alternatively, it is possible that the patients' preserved performance reflects neural plasticity that may occur in patients with pre- and perinatal hippocampal damage. Regardless, hippocampal damage generally does appear to disrupt visual WM, but more work is needed to assess whether these deficits can be compensated for if the hippocampal damage occurs very early in life.

A number of studies from our own lab have examined the role of the hippocampus in visual working memory and have suggested that the hippocampus is primarily involved in supporting familiarity-based WM discriminations, whereas recollection-based WM responses are relatively well-preserved. For example, Aly and Yonelinas (2012) showed patients with MTL damage and healthy controls two images of complex scenes and required participants to rate their confidence about whether the scenes were identical, or if one was slightly changed (i.e., pinched or expanded). Participants with MTL lesions were significantly impaired relative to controls, but, critically, ROC analysis of their confidence judgments revealed that their impairments were driven entirely by selective deficits in lower confidence familiarity-based judgments, while their high-confidence, recollection-based responses were comparable to the controls. Similar deficits were observed in the patients with selective hippocampal and more extensive MTL lesions. The results were consistent with a parallel fMRI study which indicated that familiarity-based WM responses were related to increases in hippocampal activity, where recollection-based responses were related to activity in parietal regions.

Furthermore, Goodrich and Yonelinas (2016) examined working memory for an array of colored squares in patients with MTL damage and controls who viewed the array for 300 ms, and after a 1 s delay were presented with a second array that was either identical, or had one color changed. Participants then rated their confidence for whether the color of a cued target square was the same as, or different from, its color during the first presentation. Consistent with the work of Aly and Yonelinas (2012), visual WM performance for patients was worse than controls, and ROC analysis revealed that the pattern of impairment was restricted to familiarity-based judgments, while recollection-based change-detection accuracy was comparable to controls. Critically, the pattern of results was the same for patients with broader MTL damage and those with damage restricted to the hippocampus, illustrating that hippocampal damage selectively facilitates familiarity-based working memory impairments. Moreover, in a related study, we examined WM for complex, novel objects called fribbles. Patients and controls were presented with fribbles for 500 ms, and after a 500 ms delay saw a second fribble which could be identical to the previous one, or changed either globally (i.e., the image was expanded outward or contracted inward) or discretely (e.g., one of the object features was changed). Participants then rated their confidence regarding whether the two fribbles were the same or different. Relative to controls, patients were impaired at detecting global changes to complex objects, which was expected to rely on familiarity-based WM. In contrast, patients were not impaired at detecting discrete changes to features of the object, a discrimination expected to rely on recollection, consistent with the work of Aly and Yonelinas (2012) and Yonelinas et al. (2024).

The view that the MTL is selectively involved in long-term memory and that areas outside this region are sufficient to support WM has become untenable. We now know that the MTL plays a critical role in visual perception and visual working memory, and so its role is much broader than was initially thought (Aly et al., 2013; Goodrich and Yonelinas, 2016; Goodrich et al., 2019; Konkel et al., 2008; Konkel and Cohen, 2009). In addition, within the MTL, the hippocampus and the PRC appear to support distinct processes in the sense that in LTM the hippocampus is critical in supporting recollection of specific details of the study event, whereas the PRC is sufficient to support familiarity-based discriminations. These MTL subregions also appear to support distinct processes in WM and perception, although there is still some question as to exactly what those functions are. For example, some evidence suggests that the MTL—particularly the hippocampus—may be critically important for binding or associating information in WM (Olsen et al., 2012; Yonelinas, 2013). Other evidence suggests that the hippocampus may be critical in processing scene information, whereas the PRC may be critical in processing complex object information (e.g., Lee et al., 2005a). In contrast, however, other evidence suggests that the hippocampus is particularly important for familiarity-based rather than recollection-based WM for both scenes and objects, and that this familiarity signal reflects global information about the entire study event rather than simply reflecting the memory strength of individual items within that event (Aly and Yonelinas, 2012; Yonelinas et al., 2024). Further empirical work will be needed to clarify exactly what functions these MTL regions support in visual working memory. None the less, the existing results clearly indicate that any theoretical account for MTL function needs to consider its role in both LTM and in visual WM. It is our position that the function of the hippocampus in WM is not simply binding per se, nor is it just supporting the representation of scene information, but rather it supports a global familiarity signal that binds together all the aspects that make up an event. Those events can include both scenes and visual objects—and as we describe next—the hippocampus may also be critical in supporting global familiarity for auditory events as well.

Before we consider auditory memory, we note that there has been growing interest in two potentially distinct mechanisms that underly working memory. Namely, activity silent memory based on short-term synaptic weight changes, and active maintenance of attended representations (e.g., Beukers et al., 2021; Stokes, 2015). Although future work will be needed to determine how these processes relate to recollection and familiarity, one possibility worth considering is that familiarity may reflect activity silent memory based on short-term synaptic weight changes in the hippocampus, whereas recollection reflects active maintenance of attended representations in cortical regions.

## 5 Does the medial temporal lobe contribute to auditory working memory?

Whether the hippocampus also supports auditory forms of working memory is a topic that is gaining increasing interest, though it is poorly understood at present. Historically, auditory working memory, like its visual counterpart, has been viewed largely as a cognitive process that is independent of the hippocampus, and that is supported by regions analogous to those in visual working memory. For example, Baddeley and Warrington (1970) and Cave and Squire (1992) failed to find evidence for hippocampal involvement in the digit span task, suggesting that auditory WM, too, is independent of the hippocampus. In addition, Romanski and Goldman-Rakic (2002) recorded single-neuron responses in macaque monkeys as they viewed either visual (e.g., shapes, faces, and objects) or auditory (human voices, monkey vocalizations, bells, and sirens) cues. Of the 400 recording sites, they identified 70 in the ventrolateral prefrontal cortex that selectively responded to auditory stimuli, 93% of which did not respond to visual stimuli. The localization of auditory-selective neurons to the primate PFC bears considerable resemblance to historic studies of visual WM (e.g., Goldman and Rosvold, 1970).

In humans, too, research on the processes supporting auditory WM seemed to mirror what we had learned about vision in implicating the frontal cortex rather than the MTL as being important for visual working memory. For example, Arnott et al. (2005) used fMRI to examine functional activity associated with encoding and maintenance in auditory WM for the location or identity of noise burst patterns. In their paradigm, a trial began with a verbal cue indicating which task (i.e., “location,” “identity,” and “control”) the participant was about to perform. For “location” trials, participants were instructed to remember the location of the sounds across a silent delay period; for “identity” trials they were instructed to remember the two-part noise burst pattern across the delay period, and for control trials the participants were instructed to rest. After the cue, 1 of 5 noise burst patterns was presented at 1 of 5 possible locations. Importantly, just prior to the presentation of sound 2, participants were asked one of four possible questions about the stimuli, which informed them of how they should compare the features of sound 1 to sound 2. On location trials, the questions were “which sound was most left/right/central/lateral?” while the identity trial questions were “which sound had the shortest/longest first/second beat?” This manipulation allowed the researchers to examine BOLD activity for the spatial and non-spatial tasks and compare it with BOLD responses during both the maintenance and comparison intervals, since participants would not be able to process sound 1 until they were instructed by the question, establishing a clear maintenance period. Performance across the tasks was comparable, and analysis of hemodynamic responses revealed several findings consistent with a hippocampal-independent view of auditory WM. Specifically, they noted increased parietal activations for sound localization compared to when participants had to identify the sounds, while identification was associated with greater activity in the superior temporal gyrus. Additionally, dorsolateral prefrontal, ventral prefrontal, and parietal areas displayed increased activation during the comparison stage, each of which the authors attribute to memory processes (e.g., dlPFC activity might reflect process of selecting items from memory; ventral prefrontal regions might reflect retrieval of sequences from memory). Their findings are interesting in part because they provide support for the notion that WM for audition is carried out by processes similar to those in vision, which suggests overlap between the processes which support auditory and visual WM; and, importantly, that neither depends on the hippocampus for support (Arnott et al., 2005). Similar findings have been reported elsewhere (for review, see Plakke and Romanski, 2014).

Is there any evidence that the MTL plays a critical role in auditory working memory as has been found in studies of visual working memory? Unfortunately, very few studies have directly addressed this question, however, there is some indirect evidence suggesting that it may play a role. For example, Goll et al. (2012) examined auditory perception in patients with clinically typical Alzheimer's disease (AD), and controls. They designed two auditory scene analysis (ASA) tasks: a segregation task, intended to test the ability of participants to segregate sound stimuli using timbre cues, and a grouping task, to test their ability to group temporally spaced sound stimuli into a single stream using pitch cues. Compared with the control group, the AD patients showed impairments on both the segregation and grouping tasks, further, the size of the deficits and range of performance in the AD group was similar on both tasks. Moreover, after controlling for age, gender and perceptual control task performance, there remained strong evidence for a difference in performance between AD patients and controls on both ASA tests.

The findings of Goll et al. are important for several reasons. First, they show that clinically typical AD progression is associated with impairments of auditory scene analysis (i.e., segregation and grouping). Second, they show that the deficits are not attributable to simple perceptual impairments, disease duration, or impaired executive function, that is, the deficits appear attributable to a specific deficit in auditory processing. Thus, the data show that the processes of auditory segregation and grouping are supported by neural processes that are vulnerable to Alzheimer's disease. As critically, performance on their tasks was influenced by non-verbal WM capacity, which could indicate a common mechanism by which deficits of non-verbal WM impact visuospatial and auditory information in AD. While the authors speculate that WM impairments, potentially because of a binding dysfunction, are driving their pattern of results in auditory scene analysis, of equal interest is their consideration of an ASA deficit as a symptom of AD progression. While further investigation of AD symptom progression is warranted, particularly the pre-clinical and prodromal (e.g., aMCI) stages of the disease, their findings present an interesting question: could auditory scene segregation and grouping deficits represent a symptom of disease progression, thus contributing to accurate clinical diagnosis of AD, or a potential behavioral biomarker of pre-symptomatic disease onset? Given that their pattern of results was attenuated when accounting for non-verbal WM capacity, and given that, for visual WM, the hippocampus appears to play a central role in binding stimulus associations, it seems quite reasonable to suspect that the class of impairments observed by Goll et al. in fact reflect hippocampal dysfunction associated with disease progression; though due to the fact that AD impacts regions outside the MTL, nothing definitive can be stated with regard to the role of the MTL in their study. Of course, much more work is needed to characterize the evolution of ASA impairments in relation to AD pathology, but as described next, there is evidence for a relationship between hearing loss and visual WM binding deficits in healthy adults.

In another age-related study, Loughrey et al. (2019) investigated whether age-related hearing loss (ARHL), the third most common chronic health condition in older adults, and a potentially modifiable risk factor for AD, was associated with binding deficits in visual WM. In their study, adults aged 50 and over were first given an audiometric assessment, and then binned into either the hearing loss (HL) group, or the control group. Participants studied two visual arrays and after a brief delay, were presented with a test array and required to respond whether the test array was the same as, or different from, the study array. In the “shape-only” condition, the stimuli were two random six-sided polygons, while in the “shape-color binding” condition, the stimuli were two polygons selected from the same pool of shapes, but they were assigned two colors from a set of eight possible colors. In the shape-only condition, a change occurred if one of the new (i.e., test array polygons) features replaced one of the studied features, and in the shape-color condition, changes occurred if features were swapped across items (e.g., colors at study are swapped at test). A standard neuropsychological test battery revealed only one between-group difference, on a test of visuospatial ability where the HL group performed significantly more poorly than the controls. The results of their binding task revealed no differences in performance between the hearing loss group and the control group for the shape-only condition, but the HL group demonstrated a significant drop in accuracy from the shape-only to the shape-color binding condition. Additionally, within the shape-color binding condition, the HL group performed significantly more poorly than the control group. The Loughrey study is, to our knowledge, the first to link age-related hearing loss and deficits in visual WM binding. Considered in light of the Goll et al. (2012) study showing that patients with AD were impaired at auditory scene analysis, the results suggest that additional studies examining the role of the MTL in auditory working memory may be particularly informative.

More direct evidence that the hippocampus may be involved in auditory WM comes from work by Kumar et al. (2016). In this study, during fMRI scanning, participants heard two tones, picked randomly from either a high-pitch category or a low-pitch category. After each tone was presented, the participants saw a cue instructing them to keep one of the two tones in mind for a delay of 16 s. Finally, a probe tone was presented and participants had to determine whether the probe was the same as, or different from, the maintained tone. This task design permitted the researchers to examine functional connectivity at each stage of working memory (i.e., encoding, maintenance, and retrieval). Behaviorally, participants performed the task with an accuracy ranging from 67 to 98%. Critically, analysis of hippocampal BOLD activity revealed significant activations during encoding, maintenance, and retrieval in their WM task. More specifically, the researchers observed greater activations in the anterior hippocampus at encoding, and the posterior hippocampus at retrieval. Functional connectivity analysis revealed a number of important findings. First, during encoding, as compared with maintenance, there was no long-range connectivity between the auditory cortex and either the hippocampus or inferior frontal gyrus (IFG). During maintenance, as compared with encoding, however, the auditory cortex was strongly connected to both the hippocampus and IFG. Second, during retrieval, as compared with maintenance, there was no significant difference in connectivity. Finally, during retrieval as compared with encoding, the auditory cortex was again strongly connected to the hippocampus and IFG. The authors attribute auditory cortex-hippocampal maintenance activity to the role of the hippocampus in keeping representations active by recalling them from LTM. Nonetheless, the data do suggest a role for the hippocampus in auditory WM (Kumar et al., 2016).

In addition, Dimakopoulos et al. (2022) found evidence that the auditory cortex and hippocampus do indeed communicate during WM encoding. In that study, investigators recorded scalp and intracranial EEG (iEEG) while participants heard and then rehearsed strings of letters. Specifically, sets of to-be-remembered consonants were presented in set sizes of 4, 6, or 8, followed by a brief delay. After the delay a probe letter appeared on the screen and participants were to indicate whether the probe had appeared in the previous string of consonants. In the patients with depth electrodes, measures of directed functional coupling (i.e., synchronization of electrophysiological recordings between the hippocampus and cortical regions), showed that, during encoding, information flowed from the auditory cortex to the hippocampus. During maintenance, however, the flow of information was reversed, that is, information flowed from the hippocampus to auditory cortex. The same pattern of information flow was observed in the scalp EEG participants. In short, the results of their study suggest a method by which the hippocampus and auditory cortex communicate during encoding and maintenance in auditory working memory (Dimakopoulos et al., 2022).

In sum, our understanding of the brain regions supporting auditory working memory has followed a trajectory not unlike that in visual working memory. That is, although initial neuroimaging and lesion studies provided strong evidence that regions outside the MTL such as the prefrontal and parietal cortices play a critical role in supporting WM for sounds, indirect evidence that the MTL may also play a role in auditory WM has also emerged. For example, patients with Alzheimer's disease display marked impairments in the segregation and grouping (i.e., binding) of auditory information which cannot be attributed to impaired executive function. Although, the widespread cortical atrophy observed in these patients do not allow one to draw strong conclusions about the role of MTL regions *per se*, these impairments may be related to hippocampal damage. Moreover, evidence of visual WM binding deficits associated with age-related hearing loss in cognitively normal participants suggests a link between the hippocampus, and both auditory and visual processes. In addition, more recent neuroimaging work has shown that, in auditory working memory, the hippocampus and auditory cortex are involved during the encoding, storage, and retrieval of information in auditory WM.

Going forward, studies that directly examine auditory working memory in patients with selective MTL lesions are needed to definitively determine whether these regions play a critical role in auditory working memory. In addition, it will be important to assess whether the role of the MTL in auditory WM is dependent on the binding requirements of the tasks, and whether its role may be related to recollection or familiarity-based responses, as has been observed in the visual modality. One study has examined ROCs for auditory working memory (McAnally et al., 2010). The results of that study were consistent with studies of visual WM in showing that change detection of auditory scenes produces curved, asymmetrical ROCs, reflecting the influence of familiarity and recollection, respectively. In addition, we have recently examined auditory working memory ROCs for tones and speech sounds, and found evidence for both recollection and familiarity-based responses (Hawkins et al., 2025). Our study is the first to quantify the contribution of recollection and familiarity to auditory working memory, and further extends prior research by demonstrating that the two processes are functionally dissociable in a manner analogous to visual working memory (e.g., Aly and Yonelinas, 2012). However, the extent which the hippocampus contributes to performance has not been explored.

## 6 Concluding remarks

It is now quite clear that the MTL is not limited to supporting LTM, but rather that it supports visual perception and visual working memory. Important advances have been made in characterizing the role that different regions within the MTL play in LTM and in WM. However, a number of questions still remain regarding the specific memory functions that these MTL regions play in visual WM (i.e., binding, familiarity etc.). In addition, we still do not know whether the MTL is centrally important for auditory WM processing in the same way it seems to be for visual WM.

The research reviewed in this paper suggests that the role of the hippocampus in auditory working memory may be underestimated, as was the case in the early studies of visual working memory. Further, what work has been done to elucidate the role of the hippocampus seems to suggest that it supports perceptual functions, working memory maintenance, and binding of stimulus features within and across spatiotemporal contexts. Additionally, the hippocampus is neuroanatomically connected in such a way that renders it capable of receiving and maintaining inputs from a variety of other brain areas (Aly and Turk-Browne, 2018). Such adaptability may be one of the reasons characterizing its “true” role has been such a complicated scientific endeavor. Relatedly, there is reason to suspect that additional characterization of the region's relationship to auditory function could yield promising avenues for detection of pre-clinical AD, as studies of both clinical and healthy populations have revealed deficits in both the auditory domain, and in visual WM binding, respectively, each of which may reasonably depend on normal hippocampal function. Thus, characterizing their influence on auditory working memory performance could yield important knowledge about the mechanisms which have been proposed to support working memory performance, in addition to the poorly understood links between visual binding deficits and hearing loss, as well as the link between AD progression and auditory scene analysis impairments.
